# Supplementary material for: Intraoperative Nerve Monitoring Parameters and Risk of Recurrent Laryngeal Nerve Injury in Thyroidectomy: A Systematic Review and Meta-Analysis
Source: Biomedicines. 2025 Oct 15;13(10):2516. doi: 10.3390/biomedicines13102516 (PMC12561866; doi:10.3390/biomedicines13102516)
Supplement: Supplementary file 1 [file biomedicines-13-02516-s001.zip › biomedicines-3841079-supplementary.pdf]

Table S1. The search query employed in the literature search.

Database	No	Search Query	Results
**PubMed [Date of Search 7/19/2024]**			
	#1	Dysphonia[tiab] OR "Dysphonia"[Mesh]	7014
	#2	“recurrent laryngeal nerve”[tiab] OR "Recurrent Laryngeal Nerve"[Mesh]	6780
	#3	Injur*[tiab] OR damage[tiab] OR palsy[tiab] OR palsies[tiab] OR paralysis[tiab] OR impairment[tiab] OR paresis[tiab]	2088759
	#4	#2 AND #3	4578
	#5	#4 OR #1	11365
	#6	Thyroidectom*[tiab] OR “Thyroidectomy"[Mesh]	36116
	#7	#5 AND #6	2147
	#8	Filters applied: Last 20 years + Humans only	1418
**Scopus [Date of Search 7/19/2024]**			
	#1	TITLE-ABS-KEY (Dysphonia)	15266
	#2	TITLE-ABS-KEY (“recurrent laryngeal nerve”)	11677
	#3	TITLE-ABS-KEY (Injur*) OR TITLE-ABS-KEY (damage) OR TITLE-ABS-KEY (palsy) OR TITLE-ABS-KEY (palsies) OR TITLE-ABS-KEY (paralysis) OR TITLE-ABS-KEY (impairment) OR TITLE-ABS-KEY (paresis)	4318505
	#4	#2 AND #3	8931
	#5	#4 OR #1	12520
	#6	TITLE-ABS-KEY (Thyroidectom*)	51401
	#7	#5 AND #6	3765
	#8	Filters applied: Last 20 years + Humans only	2143
**Web of Science [Date of Search 7/19/2024]**			
	#1	AB=Dysphonia	4948
	#2	AB=“recurrent laryngeal nerve”	4611
	#3	AB=Injur* OR AB=damage OR AB=palsy OR AB=palsies OR AB=paralysis OR AB=impairment OR AB=paresis	2460423
	#4	#2 AND #3	3348
	#5	#4 OR #1	8167
	#6	AB=Thyroidectom*	20359

	#7	#5 AND #6	1386
	#8	Filters applied: Last 20 years + Humans only	1000
**CENTRAL [Date of Search 7/19/2024]**			
	#1	Dysphonia	1044
	#2	“recurrent laryngeal nerve”	441
	#3	Injur* OR damage OR palsy OR palsies OR paralysis OR impairment OR paresis	158451
	#4	#2 AND #3	356
	#5	#4 OR #1	1382
	#6	Thyroidectom*	2237
	#7	#5 AND #6	213
	#8	Filters applied: Last 20 years + Humans only + Trials only	205
**Google Scholar [Date of Search 7/19/2024]**			
	With all of the words	Thyroidectomy	-
	With the exact phrase	recurrent laryngeal nerve	-
	With at least one of the words	Injury damage palsy palsies paralysis impairment dysphonia paresis	-
	Total	As per guidelines, only the first 200 records were retrieved	200

Table S2. The definition criteria of transient and permanent recurrent laryngeal nerve injury (RLNI) reported in studies examining the impact of intraoperative nerve monitoring (IONM) during thyroidectomy.

Author (YOP)	Transient RLNI Definition	Permanent RLNI Definition
**Acun (2004a)**	-	-
**Acun (2005)**	-	-
**Ahmed (2023)**	-	-
**Akici (2020)**	Vocal cord paralysis which continued for less than six months	Vocal cord paralysis which continued for more than six months
**Akkari (2014)**	-	-
**Alesina (2012)**	Recovery during 6 months after surgery	No recovery 6 months after surgery
**Al-Hakami (2019)**	-	Direct injury to the nerve and cord palsy for more than 6 months
**Alhan (2015)**	Vocal cord motility was proven normal by laryngoscopy within 6 months from surgery	Vocal cord paralysis persisting beyond 6 months
**Alharbi (2018)**	RLNI which improved within 6 months	RLNI which did not improve beyond 6 months
**Alqahtani (2023)**	-	-
**Ambe (2014)**	-	-
**Aygun (2022)**	Vocal cord paralysis improved in 12 months	VCP persisting beyond 12 months
**Barczyński (2009)**	Vocal cord paralysis improved in 12 months	VCP persisting beyond 12 months
**Barczyński (2010)**	Vocal cord paralysis improved in 12 months	VCP persisting beyond 12 months
**Barczyński (2012c)**	-	-
**Barczyński (2012d)**	VCP recovery within 6 months	VCP persisting beyond 6 months
**Barczyński (2014)**	Vocal cord paralysis improved in 12 months	VCP persisting beyond 12 months
**Bawa (2021)**	-	-
**Bergenfelz (2016)**	VCP recovery within 6 months	VCP for more than 6 months
**Bertelli (2021)**	VCP function recovery after 3-6 months	VCP for more than 6 months
**Bihain (2021)**	-	-
**Bryk (2024)**	VCP recovery within 6 months	VCP lasting more than 6-12 months
**Calò (2014a)**	VCP recovery within 12 months	VCP persisting beyond 12 months
**Calò (2014b)**	VCP recovery within 12 months	VCP persisting beyond 12 months
**Chan (2006)**	VCP recovery within 12 months	VCP persisting beyond 12 months
**Chen (2022a)**	-	-

**Chiang (2004)**	VCP recovery within 6 months	VCP for more than 6 months
**Chiang (2011)**	VCP recovery within 6 months	VCP for more than 6 months
**Chuang (2013)**	-	-
**Dedhia (2020)**	VCP recovery within 12 months	VCP persisting beyond 12 months
**Dionigi (2009)**	VCP recovery within 6 months	VCP for more than 6 months
**Dralle (2004)**	VCP recovery within 6 months	VCP for more than 6 months
**Erçetin (2019)**	VCP recovery within 12 months	VCP persisting beyond 12 months
**Farizon (2017)**	VCP recovery within 6 months	VCP for more than 6 months
**Fassari (2024)**	-	-
**Fei (2022)**	VCP recovering within 3 months	VCP persisting beyond 3 months
**Formanez (2016)**	VCP recovery within 6 months	VCP for more than 6 months
**Frattini (2010)**	-	-
**Gremillion (2012)**	-	-
**Gunn (2020)**	-	-
**Gür (2019)**	-	-
**Gutierrez-Alvarez (2023)**	-	-
**Hamilton (2019)**	-	-
**Hei (2016a)**	VCP recovery within 6 months	VCP for more than 6 months
**Hei (2016b)**	VCP recovery within 6 months	VCP for more than 6 months
**Hu (2016)**	VCP recovery within 6 months	VCP for more than 6 months
**Iqbal (2016)**	VCP recovery within 6 months	VCP for more than 6 months
**Jawad (2018)**	VCP recovery within 6 months	VCP for more than 6 months
**Joliat (2017)**	VCP recovery within 6 months	VCP for more than 6 months
**Jonas (2006)**	-	-
**Kai (2017)**	VCP recovery within 6 months	VCP for more than 6 months
**Karpathiotakis (2022)**	VCP recovery within 6 months	VCP for more than 6 months
**Khan (2022)**	VCP recovery within 6 months	VCP for more than 6 months
**Kim (2021)**	-	-
**Kuryga (2021)**	VCP recovery within 12 months	VCP persisting beyond 12 months
**Landerholm (2014)**	-	-

**Lenay-Pinon (2021)**	VCP recovery within 12 months	VCP persisting beyond 12 months
**Leow (2020)**	VCP recovery within 6 months	VCP for more than 6 months
**Ling (2020)**	VCP recovery within 6 months	VCP for more than 6 months
**Liu (2020)**	VCP recovery within 6 months	VCP for more than 6 months
**Liu (2021)**	VCP recovery within 6 months	VCP for more than 6 months
**Machens (2018)**	VCP recovery within 6 months	VCP for more than 6 months
**Mahoney (2021)**	-	-
**Maksimoski (2022)**	VCP recovery within 6 months	VCP for more than 6 months
**Marin Arteaga (2018)**	VCP recovery within 6 months	VCP for more than 6 months
**Maurer (2020)**	VCP recovery within 6 months	VCP for more than 6 months
**Messenbaeck (2018)**	-	-
**Mirallié (2018)**	VCP recovery within 6 months	VCP for more than 6 months
**Mizuno (2019)**	-	-
**Mohammad (2022)**	VCP recovery within 6 months	VCP for more than 6 months
**Moreira (2020)**	VCP recovery within 6 months	VCP for more than 6 months
**Muhammad (2021)**	VCP recovery within 6 months	VCP for more than 6 months
**Nagaoka (2022)**	VCP recovery within 6 months	VCP for more than 6 months
**Nayyar (2020)**	-	-
**Paek (2022)**	VCP recovery within 6 months	VCP for more than 6 months
**Pei (2021)**	-	-
**Périé (2013)**	VCP recovery within 6 months	VCP for more than 6 months
**Porseyedi (2012)**	-	-
**Prokopakis (2013)**	VCP recovery within 4 months	-
**Raval (2009)**	VCP recovery within 6 months	VCP for more than 6 months
**Razavi (2018)**	-	-
**Ritter (2021)**	VCP recovery within 12 months	VCP persisting beyond 12 months
**Robertson (2004)**	-	-
**Rudolph (2014)**	VCP recovery within 6 months	VCP for more than 6 months
**Russell (2021)**	VCP recovery within 6 months	VCP for more than 6 months
**Sanguinetti (2014)**	-	-
**Sarkis (2017)**	VCP recovery within 6 months	VCP recovery elay until 12 months

**Schneider (2019)**	VCP recovery within 6 months	VCP for more than 6 months
**Sena (2019)**	-	-
**Shindo (2007)**	VCP recovery within 6 months	VCP for more than 6 months
**Snyder (2010)**	-	-
**Snyder (2013)**	-	-
**Sopiński (2017)**	-	-
**Stevens (2012)**	VCP recovery within 6 months	VCP for more than 6 months
**Tabriz (2024)**	VCP recovery within 6 months	VCP for more than 6 months
**Vasileiadis (2016)**	-	-
**Velayutham (2022)**	VCP recovery within 6 months	VCP for more than 6 months
**Wojtczak (2017)**	VCP recovery within 6 months	VCP for more than 6 months
**Wu (2017)**	VCP recovery within 6 months	VCP for more than 6 months
**Wu (2018)**	VCP recovery within 6 months	VCP for more than 6 months
**Xu (2023)**	VCP recovery within 6 months	VCP for more than 6 months
**Yu (2020)**	-	-
**Yuksekdag (2019)**	VCP recovery within 6 months	VCP for more than 6 months

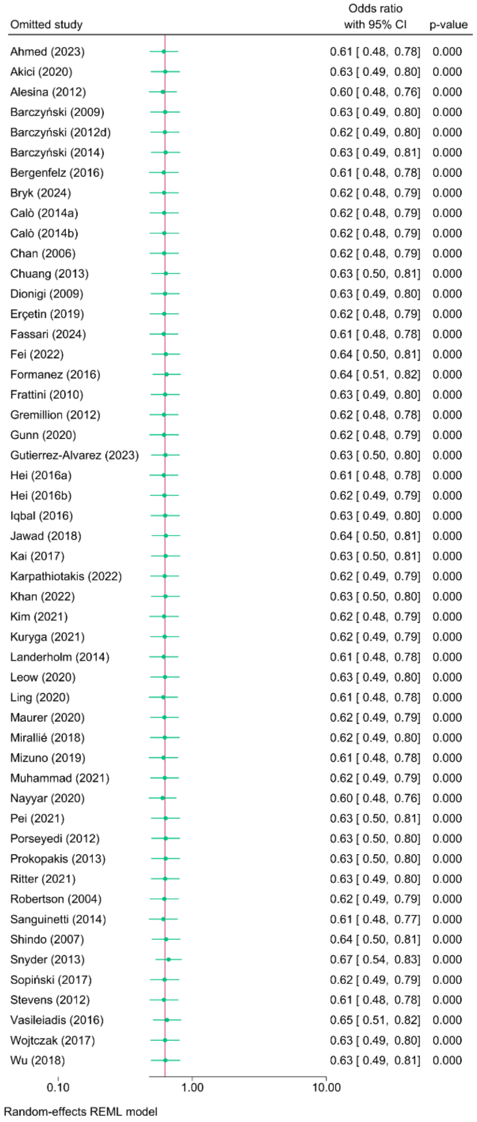

Figure S1. Leave-one-out sensitivity analysis of the direct head-to-head comparison between IONM and non-IONM regarding transient unilateral RLNI.

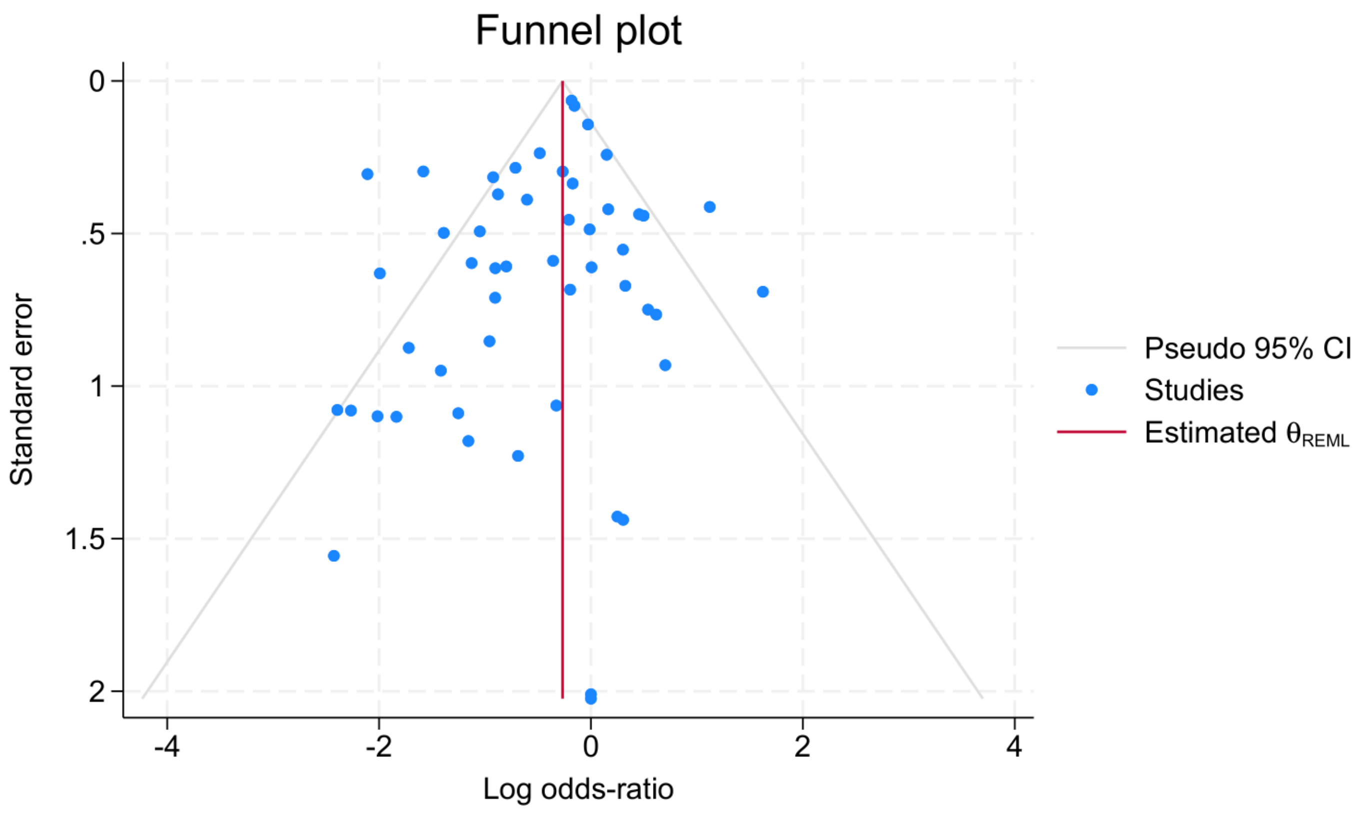

Figure S2. Funnel plot of the publication bias of unilateral transient RLNI in studies reporting the direct head-to-head comparison between IONM and no IONM.

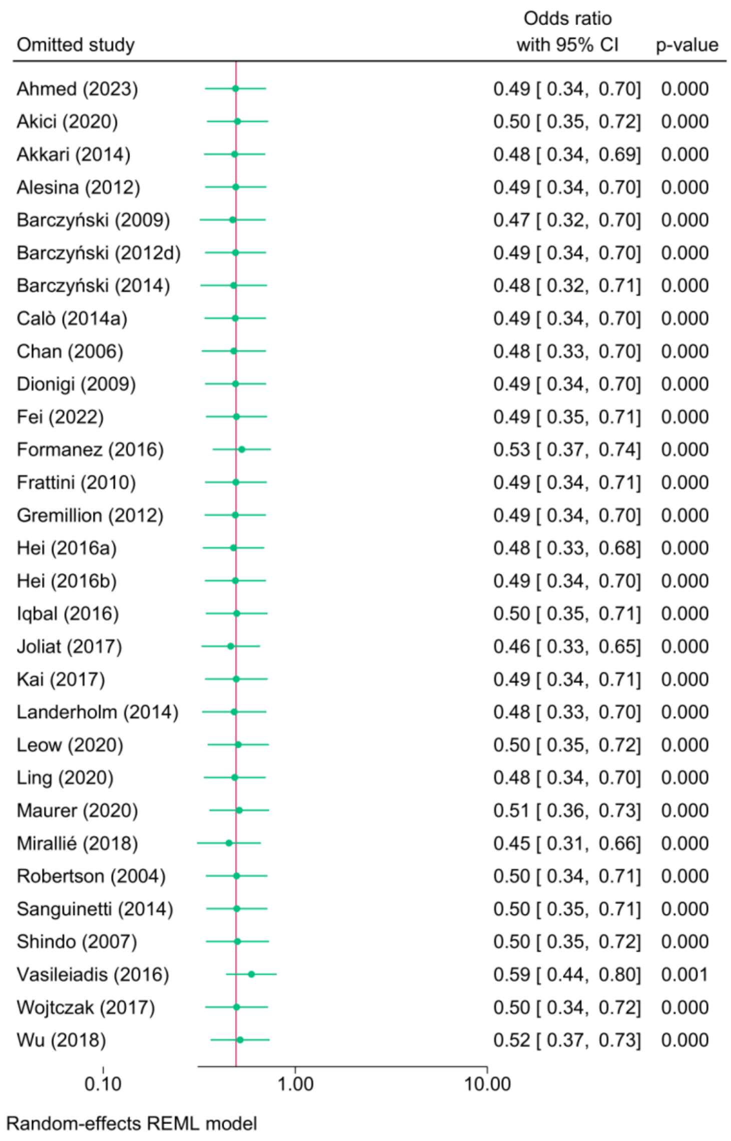

Figure S3. Leave-one-out sensitivity analysis of the direct head-to-head comparison between IONM and non-IONM regarding transient unilateral RLNI.

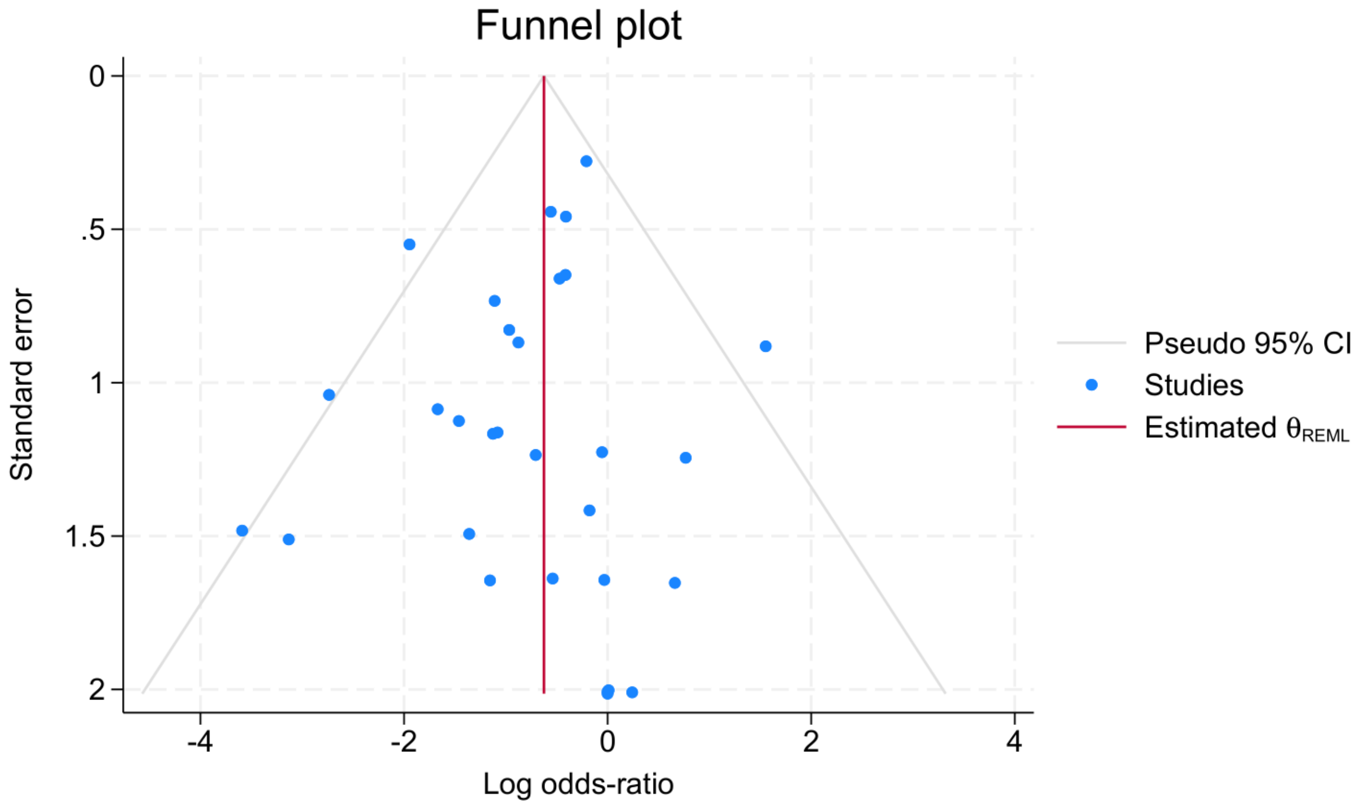

Figure S4. Funnel plot of the publication bias of unilateral transient RLNI in studies reporting the direct head-to-head comparison between IONM and no IONM.
